# Glycine Increases Insulin Sensitivity and Glutathione Biosynthesis and Protects against Oxidative Stress in a Model of Sucrose-Induced Insulin Resistance

**DOI:** 10.1155/2018/2101562

**Published:** 2018-02-21

**Authors:** Mohammed El-Hafidi, Martha Franco, Angélica Ruiz Ramírez, José Santamaria Sosa, José Antonio Pineda Flores, Ocarol López Acosta, Monserrath Chávez Salgado, Guillermo Cardoso-Saldaña

**Affiliations:** ^1^Departamento de Biomedicina Cardiovascular, Instituto Nacional de Cardiología Ignacio Chávez, Juan Badiano No. 1, Colonia Sección XVI, Tlalpan, 14080 Mexico City, Mexico; ^2^Departamento de Nefrología, Instituto Nacional de Cardiología Ignacio Chávez, Juan Badiano No. 1, Colonia Sección XVI, Tlalpan, 14080 Mexico City, Mexico; ^3^Departamento de Endocrinología, Instituto Nacional de Cardiología Ignacio Chávez, Juan Badiano No. 1, Colonia Sección XVI, Tlalpan, 14080 Mexico City, Mexico

## Abstract

Oxidative stress and redox status play a central role in the link between insulin resistance (IR) and lipotoxicity in metabolic syndrome. This mechanistic link may involve alterations in the glutathione redox state. We examined the effect of glycine supplementation to diet on glutathione biosynthesis, oxidative stress, IR, and insulin cell signaling in liver from sucrose-fed (SF) rats characterized by IR and oxidative stress. Our hypothesis is that the correction of glutathione levels by glycine treatment leads to reduced oxidative stress, a mechanism associated with improved insulin signaling and IR. Glycine treatment decreases the levels of oxidative stress markers in liver from SF rats and increases the concentrations of glutathione (GSH) and *γ*-glutamylcysteine and the amount of *γ*-glutamylcysteine synthetase (*γ*-GCS), a key enzyme of GSH biosynthesis in liver from SF rats. In liver from SF rats, glycine also decreases the insulin-induced phosphorylation of insulin receptor substrate-1 (ISR-1) in serine residue and increases the phosphorylation of insulin receptor *β*-subunit (IR-*β*) in tyrosine residue. Thus, supplementing diets with glycine to correct GSH deficiency and to reduce oxidative stress provides significant metabolic benefits to SF rats by improving insulin sensitivity.

## 1. Introduction

Oxidative stress is a mechanistic link between obesity and insulin resistance due to the high level of reactive oxygen species (ROS) generated under the effect of the increased amount of free fatty acids (FFA) and other substances delivered from fat adipocyte to peripheral tissues [1, 2]. FFA accumulation in liver results in lipotoxicity by enhancing superoxide anion generation via NADPH oxidase and mitochondrial respiratory chain [3]. The consequent increased generation of ROS and reactive aldehydic derivatives and the downregulation of genes for antioxidant enzymes induce an altered intracellular redox status causing oxidative stress and cell death, via ATP, NAD, and *γ*-glutamyl-cysteinyl-glycine (glutathione (GSH)) depletion [4]. Glutathione, the most abundant low-molecular-weight thiol, is synthetized from glutamate, cysteine, and glycine and mediates two sequential cytosolic enzymes: *γ*-glutamylcysteine synthetase (*γ*-GCS) and GSH synthetase (GS). The ratio GSH/GSSG (glutathione/oxidized glutathione) is the major redox couple in animal cells, and it is sensitive to uncontrolled H_2_O_2_ generation and to GSH biosynthesis defect.

Glutathione deficiency and its relationship with obesity and insulin resistance is controversial. Recently, depletion of glutathione by pharmacological inhibition or by genetic deletion of *γ*-GCS was reported to protect mice from diet-induced obesity and insulin resistance [5, 6]. On the other hand, glycine and cysteine, when supplemented together to the diet of aged and diabetic patients, have been described to increase glutathione bioavailability, reduce oxidative stress, and enhance glucose metabolism [7, 8]. Thus, even if GSH deficiency is well known to be related to oxidative stress, its relation to the development of insulin resistance (IR) and nonalcoholic fatty liver disease (NAFLD) is not yet well established. GSH, as a cofactor of glutathione peroxidases (GPX), participates in neutralizing H_2_O_2_ and lipid hydroperoxides, but it also provides an abundant source of reducing equivalents for disulphide bond reduction within proteins, a process involved in the regulation of several protein activities and signaling cascades within cell [9, 10]. ROS have been reported to play a major role in FFA-induced insulin resistance by phosphorylating insulin receptor substrate-1 (IRS-1) in serine residue and by inhibiting the downstream insulin signaling. N-Acetylcysteine as an antioxidant and precursor of glutathione was described to control hyperglycemia and to modulate insulin action and secretion [11]. Recently, in an experimental model of obesity induced by sucrose diet, we observed that glycine supplemented to the diet of sucrose-fed (SF) rats decreases the levels of oxidative stress markers and increases glutathione levels in vascular tissue [12]. In addition, glycine has been found to lower triglyceride and intra-abdominal fat levels in SF rats [13]. This finding allows us to hypothesize that lipotoxicity induced in rat liver by sucrose feeding may be related to oxidative stress and to decreased GSH levels which are involved in insulin resistance. Therefore, increasing glutathione biosynthesis by glycine supplementation to the diet of SF rats may protect the liver from oxidative stress and IR.

## 2. Materials and Methods

### 2.1. Animals and Their Treatment

All animal experiments were conducted in compliance with our Institution's ethical guidelines for animal research and were approved by the laboratory and animal care committee of our institution. Male Wistar rats aged 28 days and weighing approximately 65 ± 5 g were housed in collective cages (Nalgene, NY, USA) 3 rats each, under controlled temperature, and a 12-hour light-dark cycle. They were randomly separated into two groups: control and experimental. The control group (C) received tap water. The experimental group (sucrose-fed (SF) rats) received 30% commercially refined sucrose in their drinking water during a 20-week period. All animals were fed with Purina 5001 rat chow (Richmond, IN, USA.) ad libitum. After 20 weeks, each group was divided into two subgroups. The first subgroup (C) continued to drink water; the second subgroup (CG) received water supplemented with 1% glycine. In parallel, the group that had received sucrose in its drinking water was also divided into two subgroups: one subgroup (SF) continued to receive 30% sucrose in its drinking water and the other one (SFG) received sucrose supplemented with 1% glycine. The food (g/day/rat) and water (mL/day/rat) intake was monitored every 2 days during the last week of experimental period of glycine treatment. The individual caloric intake (kJ/day/rat) was assessed from the amount of food and sucrose ingested. The total number of rats used in this study was eighty (80 animals), and it was distributed according to the type of experimental procedure. For hyperinsulinemic-euglycemic clamp experiment, a group of 32 rats was destined to this assay and was divided into 4 subgroups: control, SF, and the two groups with glycine treatment (*n* = 8 rats per subgroup). Another group of 24 rats was destined to blood and tissue collection to analyze the effect of glycine on the general characteristics of animals such as lipid and intra-abdominal fat and to analyze glutathione metabolism, oxidative stress markers, and anti-oxidant enzyme content and activities (*n* = 6 rats per subgroup). Finally, the insulin sensitivity was assayed using a group of 24 rats which was divided into 4 subgroups of C, CG, SF, and SFG (*n* = 6 rats per subgroup).

### 2.2. Plasma and Tissue Sampling

After overnight fasting, the animals were decapitated; their blood was collected in tubes containing EDTA (0.1%) and centrifuged immediately at 600 ×g for 20 min at 4°C. The plasma obtained, to which 0.005% butylated-hydroxytoluene was added as antioxidant, was stored at −70°C until needed for lipid analysis. The liver was immediately washed in a physiological solution containing 0.9% NaCl in an ice bad and fragmented in several portions and frozen in nitrogen and stored at −70°C until needed for oxidative stress markers, glutathione metabolism, glutathione protein biosynthesis, antioxidant enzyme analysis, and glycine supplementation.

Plasma and liver triglyceride (TG) concentrations were measured according to the method described by Nägele et al. [14]. Plasma insulin was measured by radioimmunoassay using standard commercial kits (Linco Research Inc., St. Charles, MO). Each commercial assay was calibrated with standards from the manufacturer. Intra-abdominal fat was dissected from the retroperitoneal cavity and around both kidneys and immediately weighed. Visceral and duodenal fat was not included in this procedure.

### 2.3. Hyperinsulinemic-Euglycemic Clamp

Insulin resistance was determined by the direct hyperinsulinemic-euglycemic clamp method as described by Tran et al. [15]. After the treatment period, rats were fasted overnight and anesthetized with pentobarbital (60 mg/kg, i.p.). The left carotid artery and both left and right jugular veins were catheterized for blood collections, insulin and glucose infusions, respectively. Catheters (PE50) were exteriorized at the dorsal neck, and the external catheter ends were replugged with stainless steel tubing plugs. Catheters were flushed with 10 U/mL heparinized saline to prevent clotting. The clamps were performed in conscious animals a few days later after rats completely recovered from surgery. Rats (*n* = 8 per group), fasted for 12 h, were infused for 2 h with human insulin (Insulinex R, Pisa) by means of a high-precision peristaltic pump into the right jugular vein, at a rate of 18 mU/min/kg. The 34% glucose solution was administered to the animals by another high-precision peristaltic pump into the left jugular vein. Throughout the infusion, the carotid artery was assessed every 10 min using ACCU-CHEK active glucometer (Roche Diagnostics, Basel, Switzerland) for monitoring glucose levels during 2 hours. In this technique, the infusion of insulin at high concentration (hyperinsulinemia) increases the internalization of circulating glucose into insulin-sensitive tissues and inhibits endogenous glucose production by the liver. Plasma glucose lowering is prevented by a variable flow of glucose solution. The amount of exogenous glucose required to maintain plasma glucose at an initial level is quantified by the glucose infusion rate (GIR). GIR is a measure of the ability of insulin to enhance glucose internalization and removal of glucose production, that is, a measure of tissue sensitivity to insulin in each animal. A blood sample was taken during fasting to provide an initial value. After an adaptation period of 10 min, another blood sample was taken to measure blood glucose levels and then euglycemia was maintained by variable infusion of glucose according to the determination of plasma glucose at 10 min intervals during the two-hour clamp.

### 2.4. Insulin Signaling

The animals (*n* = 6 per group) were anesthetized with pentobarbital (60 mg/kg body weight) and submitted to surgery to access the inferior cava vein by which insulin was administered (100 nmol/kg body weight). One min later, a liver portion was excised and homogenized at 4°C using lysis buffer containing 50 mM NaF, 1% TritonX100 and 100 mM Tris-HCl, 5 mM sodium pyrophosphate, and 10 mM EDTA (pH 7.2) and supplemented with 1 mM sodium orthovanadate, 1 mM phenylmethylsulfonyl-fluoride (PMSF), 2 *μ*g/mL aprotinin, 2 *μ*g/mL pepstatin, and 2 *μ*g/mL leupeptin as antiproteases. The sample was then centrifuged at 8000 ×g for 10 min at 4°C to remove connective tissue and cell debris. The homogenate protein level was quantified using the method of Lowry [16]. Two hundred micrograms of protein of each sample was collected, suspended in 25 *μ*L of loading buffer containing 125 mM Tris-HCl (pH 6.8), 20% glycerol, 4% SDS, 10% 2-mercaptoethanol, and 0.004% bromophenol blue, completed to 50 *μ*L with Leammli solution (40 mM Tris, 1% SDS and 1% *β*-mercaptoethanol).

One hundred micrograms of protein of each sample was loaded on SDS-PAGE gel which acrylamide percentage depended on the protein to analyze as is indicated in each figure legend. The electrophoresis was run for 3 hours at 120 V. The protein transfer was performed onto the PVDF membrane (polyvinylidene difluoride, pore size 0.22 *μ*m, Millipore Corporation) at 350 mA for 20 min in a chamber of semidry transfer (Bio-Rad, Trans Blot SD). The nonspecific protein detection was reduced by blocking membranes in TBS solution containing 25 mM Tris base, 150 mM NaCl, 5% nonfat milk, and 0.1% Tween 20. The membranes were then incubated with polyclonal antibodies against IR-*β* and IRS-1 and their phosphorylated forms in the tyrosine and serine residues, respectively (Santa Cruz Corporation, CA), at a dilution of 1 : 1000 in TBS-Tween (0.1%) overnight at 4°C under gentle agitation. At the end of incubation, membranes were washed 3 times with TBS-Tween (0.1%) for 5 min each and twice with TBS for 10 min each. As a secondary antibody, anti-rabbit IgG peroxidase conjugate was used at a dilution of 1 : 1000 in TBS-Tween (0.1%) at room temperature for 2 hours. The membranes were subsequently washed 5 times, twice with TBS-Tween (0.1%), and three times with only TBS; proteins were revealed by a chemiluminescent reagent (Clarity, Bio-Rad). Membranes were exposed to image plates (BioMax, Kodak) for 5 min. The bands obtained were analyzed by an UVP image analyzer (UVP Inc., Upland, CA, USA).

### 2.5. Western Blot of Protein Involved in GSH Biosynthesis and the Antioxidant System

At the end of the treatment period, rats (*n* = 6 per group) were fasted overnight and sacrificed by decapitation. A portion of the liver was excised and homogenized at 4°C using lysis buffer containing 1% TritonX100 and 100 mM Tris-HCl and 10 mM EDTA (pH 7.2) and supplemented with 1 mM phenylmethylsulfonyl-fluoride (PMSF), 2 *μ*g/mL aprotinin, 2 *μ*g/mL pepstatin, and 2 *μ*g/mL leupeptin as antiproteases. The sample was then centrifuged at 8000 ×g for 10 min at 4°C to remove connective tissue and cell debris. The protein level was quantified according to the method of Lowry [16]. Two hundred micrograms of protein of each sample was collected, suspended in 25 *μ*L of loading buffer containing 125 mM Tris-HCl (pH 6.8), 20% glycerol, 4% SDS, 10% 2-mercaptoethanol, and 0.004% bromophenol blue, completed to 50 *μ*L with Leammli solution (40 mM Tris, 1% SDS and 1% *β*-mercaptoethanol).

Eighty micrograms of protein of each sample was loaded on SDS-PAGE gel which acrylamide percentage varies from 10 to 13% depending on the molecular weight of protein to analyze as indicated in each figure legend. The electrophoresis and protein transfer was performed as described above for insulin sensitivity. The transfer membranes (PVDF) were incubated with different antibodies against GSH metabolism proteins (*γ*-GCS and GS) and against antioxidant enzymes such as GPX-1, GPX-3, Cu/Zn-SOD, and CAT in liver homogenates. Anti-*γ*-GCS, anti-GS, anti-GPX-1 and 3, anti-Cu/Zn-SOD, and anti-CAT antibodies were raised in a rabbit (used at 1/1000) and were from Santa Cruz Corporation. For loading control, the same membrane was incubated for 30 min in a solution of 40 mM SDS and 250 mM glycine (pH 2) to remove reagents and antibodies used above. It was subsequently incubated for 1 hour in TBS-Tween (0.1%) solution containing 5% nonfat milk and then treated as described above, but anti-*β*-actin antibody (from Abcam) at 1/2000 was used in this case, and the secondary antibodies were peroxidase conjugated. Proteins were revealed by a chemiluminescent reagent (Clarity, Bio-Rad) and membranes were exposed for 5 min to image plates (BioMax, Kodak). The image plate was scanned with GelDoc-It Imaging System (UVP Inc., Upland, CA, USA). Bands were analyzed by a UVP image analyzer, and optical density was evaluated with VisionWorks LS software (UVP Inc., Upland, CA, USA).

### 2.6. Catalase, SOD, and GPX Activities

The technique using the native polyacrylamide gel is ten times more efficient than the spectrophotometric method. It can assay the activity of a single SOD isoform on polyacrylamide gel and excludes the interference coming from non-SOD molecules in crude tissue extract, which is impossible to avoid when using the spectrophotometric method. SOD activity in liver homogenate preparation was assessed previously in [17]. Briefly, 10 *μ*L of each sample (containing 2 mg/mL total protein) was loaded onto 5% staking and 8% native polyacrylamide gel bathed in 1 × Tris-glycine buffer (pH 8.3) and the proteins separated at constant current (120 V) at 4°C for 3 hours. After electrophoresis, gels were washed for 10 min with 50 mM phosphate buffer, pH 7.8, and then incubated in a solution containing 50 mM potassium phosphate (pH 7.8) containing 275 *μ*g/mL nitro blue tetrazolium (NBT), 65 *μ*g/mL riboflavin, and 0.25% tetramethylenediamine (TEMED). After 15 min incubation in the dark, the blue NBT stain for O_2_^−^ was rinsed in phosphate buffer and illuminated for 15 min with a UV light source. SOD activity appeared as clear bands on a blue background.

For catalase activity, the gel was incubated with 50 *μ*M H_2_O_2_ in double-distilled H_2_O (ddH_2_O) for 30 min. After washing the excess of H_2_O_2_ 3 times, the gel was incubated with a mixture of FeCl_3_/Na_2_Fe(CN)_4_ (1% each) for a few seconds to stain all the gel in blue-green color except where there is CAT activity. The bands obtained in each assay were analyzed by a UVP image analyzer (UVP Inc., Upland, CA, USA). The results are reported as percentage of pixels. For loading control, the gel was stained by coomassie blue.

GPx activity was measured according to the method described by Wendel [18], using tert-Butyl hydroperoxide (tBuOOH) as a substrate. The method is based on the oxidation of GSH and the reduction of tBuOOH to its corresponding hydroxide by GPX, coupled to GSSG production by glutathione reductase (GR) activity paired to NADPH consumption monitored at 340 nm in a Beckman DU-640 Spectrophotometer. The assay mixture consists of 1 mL of 50 mM phosphate buffer with 0.4 mM EDTA (pH 7.0) and contains 0.2 mg liver homogenate protein. The final concentration of reagents in the assay mixture is 0.25 mM NADPH, 2.1 mM GSH, 5 unit/mL glutathione reductase, and 300 *μ*M tBuOOH. One unit of GPX catalyzes the oxidation of 1.0 *μ*mole of GSH to GSSG per minute at pH 7.0 and 25°C. GPX activity was calculated using molar extinction coefficient for NADPH (*ε* = 6.220 mM^−1^ cm^−1^) and expressed as U/mg protein. GPX activity is calculated as NADPH oxidized/min/mg protein = [(change in absorbance at 340 nm/min for the sample test − change in absorbance at 340 nm/min for the blank) × final volume]/[0.00622 × concentration of the sample in mg/mL protein × sample volume], where 0.00622 = micromolar extinction coefficient of NADPH at 340 nm for the 1 cm path length of the quartz cuvette.

### 2.7. Analysis of Glutathione (GSH), Cysteine (Cys), *γ*-Glutamylcysteine (*γ*-GC), and Oxidized Glutathione (GSSG) by HPLC

Two 10 mg of protein samples in a 2 mL Eppendorf tube, 500 *μ*L of extraction solution containing 5 mM DETAPAC, and 200 mM methanesulfonic acid were added and stirred with a vortex for 1 min 3 times at 5 min intervals; then, the tube was allowed to stand for 10 more min in the ice, and it was centrifuged at 10000 ×g for 10 min at 4°C. The obtained supernatant was filtered through a nylon membrane with a pore size of 0.22 *μ*m, and it was injected into HPLC (Waters) equipped with a reversed phase column (250 mm × 4.6 mm) Waters Spherisorb ODS-2 and 5 *μ*m particle size, coupled to an electrochemical detector (DECADE) fitted with a working carbon electrode and with a reference hydrogen electrode at 520 mV and with UV/Vis detector at 216 nm to detect GSSG. The elution was carried out using the isocratic mobile phase of 50 mM NaH_2_PO_4_, H_2_O, and 0.05 mM of 1-octanesulfonic acid adjusted to pH 3.0 with phosphoric acid, with a constant flow of 1 mL/min. To quantify GSH, *γ*-GC, Cys, and GSSG in samples, a standard curve with a known concentration of the different substances was used.

### 2.8. Analysis of Glycine by HPLC

One portion of liver (about 100 mg) and 100 *μ*L plasma were homogenized and deproteinized in 4 volumes of ice-cold perchloric acid (5 M) and centrifuged at 10000 ×g for 10 min at 4°C. The supernageant was neutralized by NaOH (10 N) in ice and centrifuged again at 10000 ×g for 10 min at 4°C.

Fifty *μ*L of the extract was treated with 50 *μ*L of solution containing 5% (wt/*v*) ortho-phtalaldehyde (OPA) and 0.42% (*v*/*v*) of 3-mercaptopropionic acid and mixed at room temperature for 5 min and filtered through a nylon membrane with a pore size of 0.22 *μ*m. An aliquot of 20 *μ*L of each filtered sample was analyzed by HPLC equipped with LC-18-DB, 5 *μ*m, 250 × 4.6 mm (Supelco), with fluorescence detection at *λ* = 340 nm (ex) and 420 nm (em). Mobile phase A was 25 mM NaH_2_PO_4_, adjusted to pH 7.2 with NaOH, while mobile phase B was NaH_2_PO_4_/methanol/acetonitrile (50/35/15 *v*/*v*/*v*). The separation of glycine from other amino acids was obtained with a gradient program that allowed for 3 min at 0% B followed by a 50 min step that raised eluent B to 100% at a flow rate of 1 mL/min. Then, washing at 100% B and equilibration at 0% B were performed for a total analysis time of 60 min.

### 2.9. Oxidative Stress Markers

Protein carbonyls were quantified in liver homogenates prepared as described above using a modified method and reported previously [19]. Thiobarbituric acid reactive substance (TBARS) was determined in liver homogenized as described previously [20].

### 2.10. Data Analysis

Statistical analyses were performed with Sigma plot version 13 (Systat software Inc., CA). All values are expressed as means ± SD. The differences between groups were determined by one way ANOVA with post hoc Tukey test. The number of animals used for each analysis is indicated in the figure and table legends. Statistical differences were considered significant when *p* < 0.05.

## 3. Results

### 3.1. General Characteristics of Animals

Rats put on a sucrose diet for 24 weeks developed a greater accumulation of intra-abdominal fat and had increased concentration of plasma and liver triglycerides compared with control animals. Insulin levels in plasma were also significantly higher in the SF group. In terms of body weight, plasma glucose and cholesterol concentrations, no significant differences, were observed between the two groups (Table 1). Glycine treatment significantly decreased all the parameters found altered in SF rats such as plasma and liver triglycerides, insulin, and intra-abdominal fat. In control animals, glycine had no effect. In regard to FFA-found increase in both plasma and liver homogenates from SF rats, glycine supplemented to the diet significantly reduced their level to the normal level found in control animals. As for the consumption of food and drinking water, SF animals ingested less solid food than control animals. However, there was no difference in water consumption between the two groups. Treatment of animals with glycine affected neither the consumption of solid food nor that of drinking water in the two groups. Therefore, the calorie intake of SF rats was significantly higher than that of the C group (Table 1).

### 3.2. Hyperinsulinemic-Euglycemic Clamp

**Figure 1:**
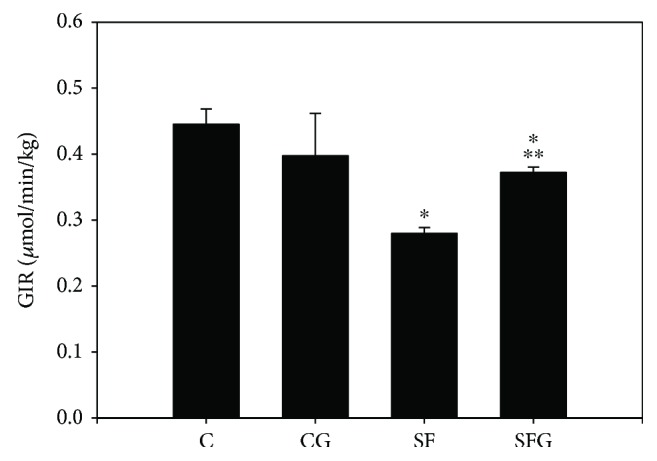
Effect of sucrose feeding and glycine treatment on glucose infusion rate (GIR) during hyperinsulinemic-euglycemic clamp in rats. Values correspond to mean ± SD (*n* = 8 different animals). ^∗^*p* < 0.05 compared to the control group; ^∗∗^*p* < 0.05 compared to the SF group. C: control; SF: sucrose-fed; CG: control + glycine; and SFG: sucrose-fed + glycine.

The insulin levels during the last 20 min clamp were not significantly different between the four dietary groups (2.7 ± 0.9 *μ*M for the C group; 2.3 ± 0.6 *μ*M for the CG group; 2.8 ± 0.7 *μ*M for the SF group; and 2.6 ± 1.2 *μ*M for the SFG group) and thus indicates that the hyperinsulinemic clamp had reached a steady state allowing us to examine the glucose infusion rate (GIR). The quantity of glucose required to establish euglycemia during this period was affected by sucrose-fed diet (Figure 1). The GIR required to maintain euglycemia was significantly lower in the SF than in the C and CG groups (0.28 ± 0.0136 versus 0.45 ± 0.007 *μ*mol/min/kg body weight). Meanwhile, glycine supplementation to the diet significantly increased GIR in the SF group but did not reach the normal level found in the C group (0.38 ± 0.008 versus 0.28 ± 0.0136 *μ*mol/min/kg body weight). Thus, the SF group was less insulin sensitive and more insulin resistant, while glycine supplementation increased insulin sensitivity and decreased insulin resistance in SF rats.

### 3.3. Glycine and Oxidative Stress Markers

Protein carbonyls are considered as markers of oxidative stress *in vivo*. According to Table 2, there was a significant increase by 150% (*p* < 0.05) in the concentration of protein carbonyls in the liver of SF animals. Glycine treatment on SF animals significantly reduced protein carbonyls but did not reach the level found in control animals. Glycine also significantly reduced protein carbonyl levels in the homogenate of liver from control animals. Regarding TBARs, a lipid peroxidation marker, glycine treatment normalized the concentration of TBARs found increased in liver homogenates from SF rats.

### 3.4. Effect of Glycine on Glutathione Biosynthesis

Table 2 shows that GSH concentration in SF animals was significantly lower by approximately 2.5-fold (*p* < 0.05) compared with that in control animals. Conversely, the amount of GSSG was significantly higher in SF animals than in control animals. Therefore the GSH/GSSG ratio significantly decreased in SF rats (*p* < 0.001) in comparison with control animals. In regard to cysteine and *γ*-glutamylcysteine, they were found significantly lower in liver from SF animals with respect to the control animals (Table 2). When glycine was supplied to SFG animals, a significant increase in the concentration of both cysteine and *γ*-GC was observed and no significant change was observed in CG rats. In all groups studied, glycine treatment significantly increased the level of GSH and did not change the concentration of GSSG in control animals without glycine treatment. In SFG rats, glycine significantly increased the concentration of GSH by 130% (*p* < 0.05) and significantly decreased GSSG. These results suggest that glycine promotes GSH biosynthesis in liver from both C and SF rats.

Table 2 also shows the concentration of glycine in plasma and liver homogenates. A significant increase in the amount of glycine was observed in SFG rats as compared with SF rats without glycine treatment. The increase of glycine in plasma and liver homogenates from CG rats did not reach a significant difference.

**Figure 2:**
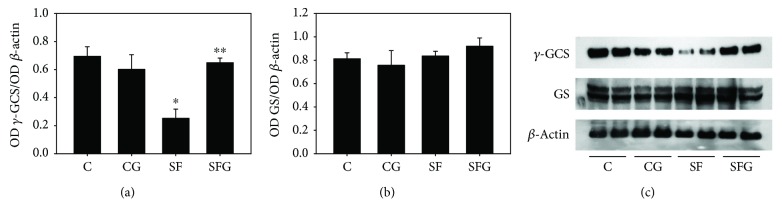
Effect of sucrose feeding and glycine treatment on *γ*-glutamylcysteine synthetase (*γ*-GCS) (a) and glutathione synthetase (GS) (b) abundance in liver homogenate of rats. (c) Representative Western blot. The OD (optical density) of each protein was normalized to the OD of *β*-actin as control load. Data are expressed as the mean ± SD (*n* = 4 different preparations from 4 different animals). ^∗^*p* < 0.05 compared to the control group and ^∗∗^*p* < 0.05 compared to the SF group.

Western blot analysis of proteins involved in the biosynthesis pathway of GSH from glutamate, cysteine, and glycine showed that the *γ*-glutamylcysteine synthetase (*γ*-GCS) level, the key enzyme in the biosynthesis of *γ*-glutamylcysteine, was decreased by approximately 2.5-fold (*p* < 0.05) in liver homogenates from SF rats as compared to control animals (Figure 2(a)). The treatment of animals with glycine completely restored the level of *γ*-GCS in SFG rats and did not change it in CG rats. In regard to GSH synthetase (GS), the enzyme involved in the biosynthesis of GSH from glycine and *γ*-glutamylcysteine did not undergo changes either in SF rats or in rats treated with glycine (Figure 2(b)).

### 3.5. Glycine and Antioxidant Enzymes

**Figure 3:**
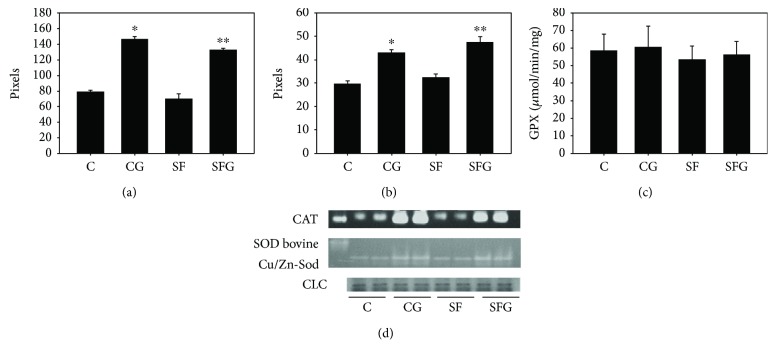
Analysis of CAT (a), Cu/Zn-SOD (b), and GPX (c) activities and a representative gel of both enzyme activities by gel staining as described in Materials and Methods. (d) Representative Western blot. The band densities were analyzed by UVP image analyzer, and the results are reported as pixels. For loading control, another gel was dedicated to be stained by coomassie blue. For the gel activity staining, data are expressed as the mean ± SD (*n* = 4 different preparations from 4 different animals). For the GPX activity assessed by spectrophotometry (see Materials and Methods), data corresponds to mean ± SD of 6 different preparations from 6 different animals. ^∗^*p* < 0.05 compared to the control group and ^∗∗^*p* < 0.05 compared to the SF group.

The technique used to assay SOD, catalase, and GPX activities, a combination of native polyacrylamide gel electrophoresis, and activity staining allowed a good resolution of SOD and CAT from other proteins which can interfere with enzyme activity in the spectrophotometry assay described elsewhere [17]. Figures 3(a) and 3(b) show that the densities of bands corresponding to the activities of CAT and Cu/Zn-SOD, the most important among antioxidant enzymes that catalyze the degradation of H_2_O_2_ to H_2_O, and the dismutation of superoxide anion to H_2_O_2_, respectively, appeared to be significantly higher in liver homogenates from of treated with glycine than those from without glycine treatment. However, the GPX activity (Figure 3(c)) assessed by a spectrophotometry method to monitor the decline of the absorbance of NADPH at 340 nm as described in Materials and Methods showed no significant difference between C and SF rats nor after glycine treatment.

**Figure 4:**
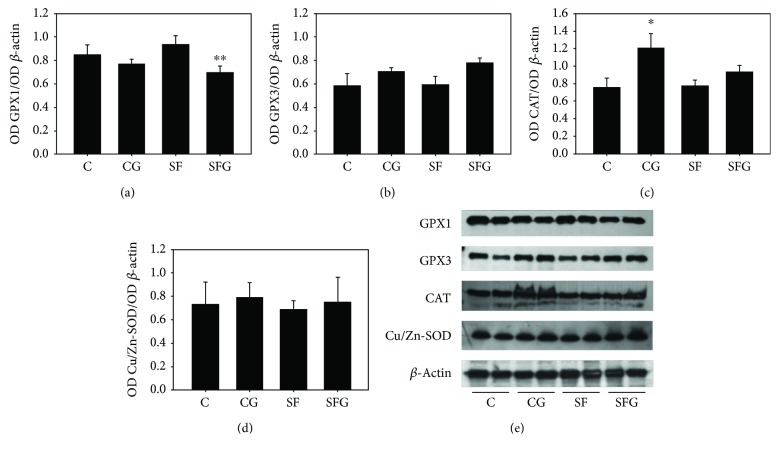
Representative Western blot and graph data (upper panels) depicting antioxidant protein abundance such as GPX1 (a), GPX3 (b), CAT (c), and Cu/Zn-SOD (d) in liver homogenate isolated from overnight-fasted C (control), SF (sucrose-fed), CG (control + glycine), and SFG (sucrose-fed + glycine) rats. Values in the panels correspond to the ratio OD of GPX1(a) or GPX3/OD of *β*-actin (b) and to the ratio OD of CAT/OD of *β*-actin (c) and to the ratio OD of CuZn-SOD/OD of *β*-actin (d). (e) Representative Western blot. Data are expressed as the mean ± SD (*n* = 4 different preparations from 4 different animals). ^∗^*p* < 0.05 compared to the control group; ^∗∗^*p* < 0.05 compared to the SF group.

Western blot analysis of GPX1 and GPX3, ubiquitous cytosolic and extracellular enzymes, respectively, which convert H_2_O_2_ to H_2_O using reduced glutathione as a cofactor, showed no significant difference between C and SF livers whereas glycine treatment decreased the GPX1 level (Figure 4(a)) and induced a moderate increase in GPX3 abundance without reaching a statistically significant difference (Figure 4(b)) in both the C and SF livers. Glycine supplemented to the diet also significantly increased the amount of CAT in liver from control rats, and only a moderate increase was observed in SF rats without reaching a statistically significant difference (Figure 4(c)). In regard to Cu/Zn-SOD, Western blot showed no difference in the protein amount between C and SF rats nor in rats treated with glycine (Figure 4(d)).

### 3.6. Effect of Glycine on Insulin Signaling

The activation of insulin receptor IR-*β* and downstream signal IRS-1 was assessed in the liver by acute administration of insulin to C and SF rats with and without glycine treatment.

**Figure 5:**
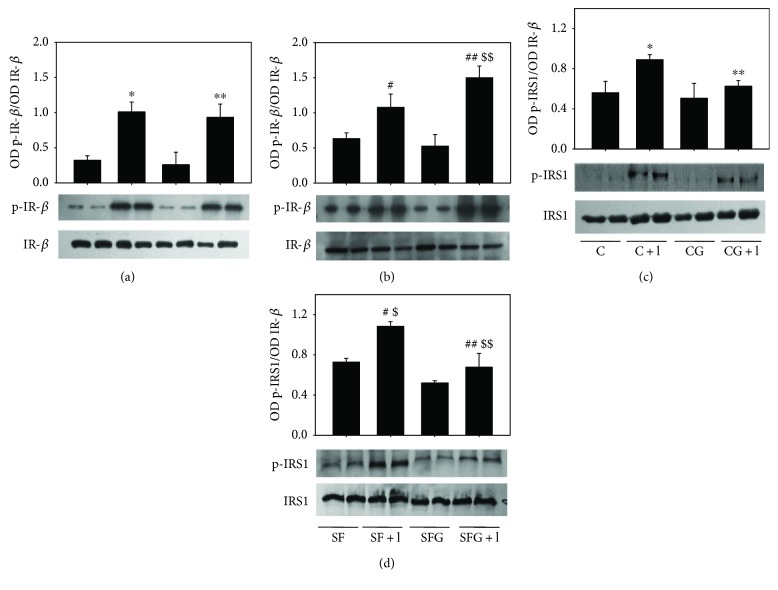
Effect of glycine treatment on the IR-*β* and IRS1 phosphorylation (p-IR-*β* and p-IRS1) in tyrosine and serine residues induced by acute insulin administration (100 nmol/kg body weight) and of total IR-*β* and IRS1 in rat liver homogenates from both C and SF rats. Values in the panels correspond to the ratio OD of p-IR-*β*/OD of total IR-*β* (a, b) and to the ratio OD of p-IRS1/OD of total IRS1 (c, d). Data are expressed as the mean ± SD (*n* = 4 different rats). ^∗^*p* < 0.05 compared to the C group and ^∗∗^*p* < 0.05 compared to the CG group. ^#^*p* < 0.05 compared to the SF group and ^##^*p* < 0.05 compared to the SFG group. ^$^*p* < 0.05 compared to the C + I group and ^$^^$^*p* < 0.05 compared to the SF + I, C + I (control + insulin), SF + I (sucrose-fed + insulin), CG + I (control + glycine + insulin), and SFG + I (sucrose fed + glycine + insulin) groups.

The autophosphorylation of IR-*β* in tyrosine residue is the first event of the signaling cascade induced by the interaction between insulin and its IR-*β*. Figures 5(a) and 5(b) show that there was no difference in the IR-*β* phosphorylation in tyrosine residue, induced by acute administration of insulin in liver from C and SF rats and glycine treatment increased the insulin-induced phosphorylation of IR-*β* in SFG animals (Figure 5(b)). In the same way, IRS-1 phosphorylation in serine residue was significantly increased in liver from SF animals as compared with control animals (Figures 5(c) and 5(d)) and glycine treatment significantly reduced the insulin-induced phosphorylation of IRS-1 in serine residue in both CG and SFG livers (Figures 5(c) and 5(d)).

## 4. Discussion

This work shows that sucrose feeding decreases GSH concentration and causes a consequent oxidative stress and insulin resistance in SF animals and that glycine supplemented to diet increases GSH biosynthesis, reduces oxidative stress, and improves insulin sensitivity and insulin cell signaling in liver from SF rats. GSH/GSSG is an important index to determine the redox status and the balance between producing and neutralizing free radicals in liver injury [21]. Thus, the ratio of GSH to GSSG, which is about 100 : 1 in the liver because of the high metabolic activity, can be influenced by uncontrolled ROS generation and by the defect in GSH biosynthesis. In this study, the decreased GSH/GSSG is related to both increased oxidative stress markers and altered GSH biosynthesis in SF liver. Several studies using animal models and the strategy of GSH depletion by buthionine sulfoximine as an inhibitor of *γ*-GCS demonstrated an increase in oxidative stress markers in the liver and various other organs *in vivo* [22], and the treatment of cells with GSH or N-acetylcysteine limits such damage and renders cells or tissues less susceptible to oxidative stress [23]. As glycine has no direct antioxidant property, the decreased oxidative markers in SFG rat liver may be mediated by an increased level of GSH, which acts as a cofactor of GPX to catalyze the conversion of H_2_O_2_ to H_2_O. In liver from SFG rats, the enhanced GSH level can also be explained by an increased amount of *γ*-GCS, a key enzyme in the biosynthesis of *γ*-glutamylcysteine and GSH. In our SF rats, the decreased amount of *γ*-glutamylcysteine level is associated with impaired *γ*-GCS expression that can be attributed to insulin resistance because insulin has been described to enhance *γ*-GCS transcription or activity in hepatocytes [24]. Thus, glycine, by improving insulin sensitivity, may increase *γ*-GCS expression that promotes the biosynthesis of *γ*-glutamylcysteine found higher in liver from SFG rats. In addition, *γ*-GCS has more control on the GSH biosynthesis pathway than GS (glutathione synthetase) which is not affected either by sucrose diet or by glycine treatment. The biosynthesis of *γ*-glutamylcysteine appears to be dependent on the availability of cysteine and glutamate. However, both amino acids can be derived from intracellular protein hydrolysis and/or from endogenous synthesis from methionine in the case of cysteine and other molecules in the organism [25, 26]. In the case of glycine, it becomes a limiting factor for GSH biosynthesis when hepatic glycine oxidation is enhanced in response to high levels of glucagon or diabetes [27] and in response to protein malnutrition [28, 29]. In SF rats, glycine found significantly decreased in liver homogenate may contribute to impaired GSH biosynthesis. Thus, the addition of glycine to the animal diet rises glycine availability in both plasma and liver homogenates and promotes the biosynthesis of GSH.

The decreased GSH, *γ*-glutamylcysteine, and cysteine levels in SF liver homogenate can also be due to their susceptibility to the oxidation by free radicals and ROS generated in liver from SF rats. Free radicals and ROS are well known to oxidize thiol groups within several biomolecules and modify their functions [30]. In a previous study, mitochondria from liver of SF rats were found to generate H_2_O_2_ with a higher rate than mitochondria from C rats [17]. H_2_O_2_ resulting from the dismutation of superoxide anion (O_2_^−^) by SOD is generally neutralized by CAT and glutathione peroxidases (GPXs). However, the lack of difference in the abundance and activities of CAT, SOD, and GPX between C and SF livers allows us to speculate that the increased oxidative stress markers in liver homogenates are due to the overproduction of ROS instead of CAT, SOD, and GPX as major enzymes of the liver antioxidant system. The increasing effect of glycine intake on CAT and Cu/Zn-SOD activities may be responsible for decreasing the levels of oxidative stress markers in SFG probably by neutralizing ROS generated at high levels in liver from SF rats. The mechanism by which glycine increases the activity of both CAT and Cu/Zn-SOD is not established and needs further experiments to elucidate it. Nevertheless, glycine by restoring the level of GSH may protect against ROS overproduction and improves antioxidant enzyme activities. Indeed, antioxidant enzymes, CAT, SOD, and GPX activities, found lower in a model of GSH depletion by buthionine sulfoximine, an inhibitor of *γ*-GCS, are restored when glutathione monoester is administrated to the model [31] and this effect may not be mediated by the induction of antioxidant enzyme expression. Indeed, when human umbilical vein endothelial cells, pretreated with buthionine sulfoximine, are incubated for 24 h with diethyl maleate to increase intracellular GSH, the mRNAs encoding Cu/Zn-SOD, CAT, and GPX are not affected [32]. Our present finding is in good agreement with the two works described above because glycine, by increasing GSH, enhances the activity of the same enzymes without a significant change in their abundance. To our current knowledge, there is no data about the direct effect of glycine nor glutathione on the activity of antioxidant enzymes. However, the endogenous glutathione system is considered of central importance in redox signaling as an efficient reducing agent to modulate cellular redox status including enzyme activities.

The glycine effect, by increasing Cu/Zn-SOD and catalase activities, may protect against ROS overproduction and improve insulin sensitivity in liver from SFG rats. The dysregulation of insulin sensitivity in SF rats may occur at high concentration of H_2_O_2_, because hydrogen peroxide, in the presence of hyperinsulinemia, was reported to have a significant negative effect on insulin signaling and to cause a selective phosphorylation in serine/threonine and loss of IRS1 and 2 proteins which further inhibit insulin response in mammalian skeletal muscle [33, 34]. Moreover, mitochondrial catalase in transgenic mice and endogenous catalase protect against high levels of H_2_O_2_ generation and delay high-fat diet-induced liver injury by improving glucose tolerance and insulin sensitivity in mice [35, 36]. Nevertheless, H_2_O_2_ at physiological concentration is exclusively regulated by GPXs and has been described to mediate signaling regulation of glucose metabolism [37]. GPXs have a greater affinity for H_2_O_2_ (Km ≈ 1 *μ*M) than catalase (Km ≈ 1 mM) and are considered the principal enzymes responsible for eliminating H_2_O_2_ that typically occurs in the low micro- and submicromolar range. In our study, the reduced effect of glycine on GPX1 and the increased activities of CAT and Cu/Zn-SOD may be related to enhanced insulin signaling and sensitivity and reduced fat accumulation and hyperlipidemia. Mice lacking GPX1 were found protected from high-fat diet-induced insulin resistance and have a reduced fasting blood glucose and improved glucose tolerance [38]. Moreover, the overexpression of GPX1 in mouse increases body weight and fat content, hyperglycemia, and hyperinsulinemia and reduces insulin sensitivity [39].

Generally, the mechanism proposed for the relationship between insulin resistance and oxidative stress in animals and cells involves the interaction of ROS with a key thiol group of signaling proteins perturbing cellular signaling and interfering with their normal function [40]. Nevertheless, oxidative stress and H_2_O_2_ were described to activate multiple stress-sensitive Ser/Thr kinases such as JNK, ERK1, and IKKb (inhibitor of NF-*κ*B), and p70S6k, which take part in insulin resistance by phosphorylation of IRS1 in serine instead of tyrosine residue, blocking insulin signaling [41]. In our SF rats, the increased phosphorylation of IRS1 in serine residues by acute insulin administration suggests alteration in the liver insulin signaling pathway by increased oxidative stress. The improved effect of glycine on IR-*β* and IRS1 phosphorylation in tyrosine and serine residues, respectively, observed in SF liver may be related to the protective effect of glycine against oxidative stress and raised GSH concentration in SFG liver. GSH was reported to protect from insulin resistance and to improve mitochondrial FFA oxidation in aging whereas chronic GSH deficiency in old mice and elderly humans was reported to be associated with decreased mitochondrial FFA oxidation and insulin resistance [42]. Furthermore, GSH is necessary for the hepatic action of insulin-sensitizing agents in regulating lipid, glucose, and amino acid utilization [43]. The glycine effect on insulin resistance in SF rats can also be related to its decreased effect on liver free fatty acid level which is well known to induce lipotoxicity and promote ROS generation in excess and mitochondrial dysfunction [17]. FFAs or their metabolites such as ceramides, diacylglycerols, and acyl-CoA were also reviewed to activate PKC*θ*, leading to increased IRS1/2 phosphorylation in serine/threonine residues instead of tyrosine residue, thus causing an imbalance in the action of insulin in liver as well as in heart and skeletal muscles [44–46]. In addition, GSH has been described to protect hepatocytes by suppressing palmitate-induced lipid peroxidation and cytotoxicity and endoplasmic reticulum stress [47].

In summary, the main finding of this study is that glycine decreases insulin resistance and restores *γ*-GCS to normal evels in SFG liver and this results in the correction of GSH deficiency induced by sucrose diet providing a significant metabolic benefit in SF rats such as protection against the oxidative stress-increased insulin sensitivity. In addition, the decreased levels of GSH raise the possibility that its availability when reduced may limit the insulin action through the enhancement of oxidative stress in SF rats. In the same time, the insulin-signaling pathways involving IR-*β*/IRS may mediate the insulin regulation of *γ*-GCS expression and GSH biosynthesis. This results in a vicious circle between insulin resistance and GSH availability, which makes our study limited regarding the direct mechanistic link between glycine availability and insulin sensitivity. Therefore, further studies are needed to distinguish between glycine and glycine-induced GSH on insulin sensitivity by combining glycine and GSH biosynthesis inhibitors in the diet of animals.

## Figures and Tables

**Table 1:** General characteristics of the animals.

Variables	C	CG	SF	SFG
Food intake (g/day/rat)	22.7 ± 0.8	19.3 ± 1.5	12.5 ± 0.7^∗^	10.7 ± 1.3
Water intake (mL/day/rat)	45.7 ± 2.7	46.5 ± 2.8	47.5 ± 2.9	44.5 ± 2.5
Energy intake (kcal/day/rat)	315.6 ± 8.5	305.5 ± 9.6	360.1 ± 8.4^∗^	356.7 ± 5.8
Body weight (g)	448 ± 12	467 ± 15	475 ± 10	466 ± 9
Intra-abdominal fat (g)	6.4 ± 1.4	6.3 ± 1.9	23.4 ± 1.8^∗^	9.8 ± 1.9^∗∗^
Plasma TG (mg/dL)	43.1 ± 7.5	32.8 ± 4.3	96.9 ± 7.1^∗^	75.0 ± 7.0^∗∗^
Liver TG (*μ*mol/mg)	77.5 ± 8.5	85 .5 ± 9.5	217.7 ± 28.4^∗^	155.7 ± 35.6^∗∗^
Plasma FFA (mM)	0.7 ± 0.07	0.5 ± 0.1	1.2 ± 0.1^∗^	0.7 ± 0.1^∗∗^
Liver FFA (*μ*mol/mg)	78.5 ± 11.4	68.6 ± 2.5	117.5 ± 27.6^∗^	62.5 ± 31.7^∗∗^
Chol (mg/dL)	38.4 ± 12.8	47.9 ± 12.1	42.2 ± 13.2	55.1 ± 16.9
Glucose (mM)	6.0 ± 1.0	5.5 ± 0.7	5.9 ± 1.2	6.4 ± 1.5
Insulin (pΜ)	111.6 ± 23.4	119.9 ± 28.4	187.5 ± 36.5^∗^	122.9 ± 19.6^∗∗^

Chol (cholesterol), glucose, and insulin were determined in plasma. TG (triglycerides) and FFA (free fatty acids) were determined in both liver homogenate and plasma. Values represent mean ± SD, *n* = 6 different animals. ^∗^*p* < 0.001 (C versus SF) and ^∗∗^*p* < 0.05 (SFG versus SF).

**Table 2:** Oxidative stress markers and glutathione (GSH) metabolism in rat liver.

Variables	C	CG	SF	SFG
GSH	45.5 ± 7.1	87.7 ± 7.9^∗^	17.4 ± 4.12^∗∗^	38.7 ± 8.4^∗∗∗^
GSSG	0.7 ± 0.3	0.87 ± 0.25	2.2 ± 0.8^∗∗^	1.17 ± 0.35^∗∗∗^
Cys	17.6 ± 4.6	20.0 ± 6.4	7.2 ± 3.4^∗∗^	23.3 ± 5.9^∗∗∗^
*γ*-GC	11.3 ± 2.7	17.7 ± 4.2	4.60 ± 2.1^∗∗^	12.9 ± 5.1^∗∗∗^
GSH/GSSG	105.6 ± 24.6	141 ± 32.5^∗^	8.91 ± 5.41^∗∗^	46.2 ± 23.5^∗∗∗^
Protein carbonyls (nmol/mg)	0.13 ± 0.03	0.33 ± 0.2	1.54 ± 0.8^∗∗^	0.6 ± 0.14^∗∗∗^
TBARs (nmol/mg)	10.4 ± 2.2	13.5 ± 3.5	23.0 ± 1.2^∗∗^	15.65 ± 3.5^∗∗∗^
Glycine				
Plasma (*μ*M)	234.6 ± 13.5	275.4 ± 24.5	205 ± 21.5	275.4 ± 15.7^∗∗∗^
Liver homogenate (nmol/mg protein)	655.7 ± 25.7	756.6 ± 35.4	584.3 ± 35.6^∗∗^	656.5 ± 37.5^∗∗∗^

Values represent mean ± SD, *n* = 6 different animals. ^∗^*p* < 0.05 (C versus CG), ^∗∗^*p* < 0.05 (SF versus C), and ^∗∗∗^*p* < 0.05 (SFG versus SF). GSSG: oxidized glutathione; Cys: cysteine; *γ*-GC: *γ*-glutamylcysteine.

## Abbreviations

ATP: Adenosine triphosphates
CAT: Catalase
FFA: Free fatty acids
GIR: Glucose infusion rate
*γ*-GCS: *γ*-Glutamylcysteine synthetase
*γ*-GC: *γ*-Glutamylcysteine
GPX: Glutathione peroxidase
GSH: Glutathione
GS: Glutathione synthetase
GSSG: Oxidized glutathione
IR: Insulin resistance
IR-*β*: Insulin receptor *β*-subunit
IRS: Insulin receptor substrate
NAD: Nicotinamide adenine dinucleotide
NADPH: Nicotinamide adenine dinucleotide phosphate hydrogen
NAFLD: Nonalcoholic fatty liver disease
ROS: Reactive oxygen species
SOD: Superoxide dismutase
C: Control
CG: Control + glycine
SF: Sucrose-fed
SFG: Sucrose-fed + glycine.
